# Neonatal Necrotizing Enterocolitis

**Published:** 2012-04-01

**Authors:** Vivek Gharpure

**Affiliations:** Department of Pediatric surgery, Children’s Surgical Hospital 13, Pushpanagari, Aurangabad, 431001, India

## QUESTIONS

1.What is the classical triad for the diagnosis of neo­natal necrotizing enterocolitis (NNEC)?

2.What etiological factors are considered significant in development of NNEC in newborns?

3.What are differential diagnoses of NNEC?

4.What are the features of Pneumatosis intestinalis in a baby with NNEC?

5.What is the significance of Portal Vein Gas in NNEC?

6.What are the indications of surgical interventions?

7.What is the role of peritoneal drainage in the man­agement of NNEC?

8.What is an appropriate postoperative care for these neonates?

9.What is the mortality and morbidity associated with this entity?

10.What are the likely complications in survivors of NNEC?

## ANSWERS

1.Abdominal distension, blood in stool, and pneumatosis intestinalis on x-ray are considered the classical features of NNEC [1].

2.Prematurity- immunological immaturity and immunological dysfunction; birth hypoxia; early feeding; hyperosmolar feeding; infection; xanthine drugs; Patent Ductus Arteriosus – babies treated for PDA with drugs or who undergo ligation have a higher incidence of NNEC; Placental insufficiency- hypertension or diabetes in mother; Blood transfusions- babies who receive transfusion have a higher incidence of NNEC [1].

3.Intestinal volvulus; Hypoplastic left heart syndrome; Intestinal malrotation; Omphalitis; Urinary tract infection; Bacterial meningitis; Neonatal sepsis; Prematurity [1,2].

4.Pneumatosis intestinalis is a radiologic sign pathognomonic of necrotizing enterocolitis (NEC). It appears as a characteristic train-track lucency configuration within the bowel wall. Intramural air bubbles represent gas produced by bacteria within the wall of the bowel. Analysis of gas aspirated from these air bubbles reveals that it consists primarily of hydrogen, suggesting that the bubbles are caused by bacterial fermentation. Carbohydrate (often lactose) fermentation by intestinal flora yields hydrogen and carbon dioxide and a series of short-chain organic acids, which can promote inflammation. Pneumatosis is present in 70%-80% of patients with NEC, although it may be fleeting or intermittent and is often an early finding. The extent of gas is not correlated with the severity of disease, nor is it specific to NEC. Pneumatosis is also seen in Hirschsprung’s disease, severe diarrhea, carbohydrate intolerance, and inspissated milk syndrome. [1].

5.Portal gas appears as linear, branching areas of decreased density over the liver shadow and represents air present in the portal venous system. Its presence is considered to be a poor prognostic sign. Portal gas is much more dramatically observed on ultrasonography. Although once heralded as an ominous sign in NEC, portal gas is now believed to be less so. It is caused by gas produced by bacteria in the portal veins or by the transmigration of gas from the bowel wall to mesenteric veins and into the portal vein. It is frequently a transient finding; the pattern is demonstrated in only 9-20% of infants with NEC [1,2].

6.Indication for operative intervention in necrotizing enterocolitis (NEC) is perforated or necrotic intestine. Infants with necrotic intestine are identified based on various clinical, laboratory, and radiologic findings. Important predictor of intestinal necrosis indicating a need for operative intervention is pneumoperitoneum. Other relative indications for operative intervention are erythema in the abdominal wall, gas in the portal vein, and positive paracentesis. Surgery is generally indicated in medically treated patient whose clinical condition deteriorates. The signs of deterioration include worsening abdominal examination findings, signs of peritonitis, worsening and intractable acidosis, persistent thrombocytopenia, rising leukocytosis or worsening leukopenia, and hemodynamic instability. Note that evaluation by a pediatric surgeon early in the course of NEC is important to avoid any delay in operative intervention. Many infants may have isolated perforations or necrotic tissue that wall off the abdominal cavity and do not show free intraperitoneal air. Knowing whether these infants may benefit from early operative intervention is difficult [1,3].

7.Neonates who are extremely ill and unable to tolerate surgery may be treated by means of peritoneal drainage. The procedure was initially intended as a means of temporizing with regard to surgical treatment, and indeed, some infants survived with this procedure alone and did not require subsequent laparotomy. A multicenter, randomized clinical trial failed to show a significant difference in survival at 90 days between primary peritoneal drainage and laparotomy with resection for premature infants with very low birth weight (less than 1500 g) and perforated NEC. Critically ill newborns with a relative contraindication to formal operative exploration may be treated with the placement of a peritoneal drain. Although this is typically a temporizing measure, these extremely ill infants may recover with drain placement alone and do not require exploratory laparotomy. Peritoneal drain placement may be the treatment of choice for extremely small (less than 600 g) premature newborns. Such premature, critically ill infants cannot tolerate formal exploration, and drain placement may be preferred and definitive. Nevertheless, many infants whose condition is too unstable for formal exploration do not survive, regardless of interventions [3].

8.After undergoing an operation for NEC, infants should continue to receive intravenous antibiotics and total parenteral nutrition for at least 2 weeks. Supportive care, including ventilatory support, fluid and electrolyte monitoring and replacement, and correction of anemia and coagulopathy, should continue. During surgery infants with NEC often develop a coagulopathy that continues after surgery and can be difficult to manage. Blood can fill the abdominal cavity rapidly and create a compartment syndrome that requires drainage. Any infants with continued clinical deterioration must be evaluated for residual intestinal gangrene and possibly repeat surgical exploration. Infants who improve postoperatively should not resume enteral feedings for at least 10-14 days [1].

9.Mortality rate in NEC ranges from 10% to more than 50% in infants who weigh less than 1500 g, compared with a mortality rate of 0-20% in babies who weigh more than 2500 g. Extremely premature infants (1000 g) are still particularly vulnerable, with reported mortality rates of 40-100%. One study comparing mortality rates for term versus preterm infants reported rates of 4.7% for term infants and 11.9% for premature babies [1-3].

10.Stricture; Short bowel syndrome; Cholestatic liver disease; Recurrence; Neurodevlopmental disorders; Vascular access/catheter related complications [1,2].

## Footnotes

**Source of Support:** Nil

**Conflict of Interest:** None declared

